# Sedation for Colonoscopy Procedures Using Dexmedetomidine Versus Propofol-Fentanyl Infusions: A Prospective Randomized Controlled Trial

**DOI:** 10.4274/TJAR.2024.231485

**Published:** 2024-05-03

**Authors:** Sameh Hamdy Seyam, Mohamed Abdelgawad Abdelhalim Aboelsuod, Ismail Mohamed Abdelgawad Ahmed, Abdallah Elabd Hassan

**Affiliations:** 1 Al-Azhar University Faculty of Medicine, Department of Anaesthesiology, Intensive Care and Pain Management, Cairo, Egypt

**Keywords:** Colonoscopy, dexmedetomidine, outpatient sedation, perioperative care, propofol, sedation

## Abstract

**Objective:**

Different anaesthetists for sedation or monitored anaesthesia care have been used for colonoscopy. The target of this research was the ability to perform colonoscopy under a painless degree of sedation and the prevalence of undesired proceedings.

**Methods:**

A total of 60 patients were randomly divided into two groups: Group D received dexmedetomidine and Group PF received propofol-fentanyl. Patients in both groups received the same infusion ratio. The minimum infusion amount of dexmetatomidine is (0.1 to 0.4 μg kg^-1^ h^-1^) in Group D, whereas fentanyl is administered at a rate of 0.01 to 0.05 μg kg^-1^ min^-1^ in the PF group during the approximately 45-min colonoscopy.

**Results:**

Group D exhibited significantly lower modified Observer’s Assessment of Alertness/Sedation (OAA/S) scores at intraoperative time points T1-T12. Group D also exhibited significantly lower visual analog scale scores for pain at intraoperative time points T4 and T7. The mean arterial pressure was significantly lower in Group D at intraoperative times T6-T8 and T11-T12, as well as upon admission to the post-anaesthesia care unit (PACU) and 30 min after admission to the PACU. The results of the ANOVA tests revealed a significantly lower heart rate in Group D. The respiratory rate exhibited a notable decrease during time intervals T8 and T10 in the PF group.

**Conclusion:**

The administration of dexmetatomidine and propofol-fentanyl during colonoscopy was found to be safe. In addition, dexmetatomidine may present significant benefits in this context because of its lower occurrence of adverse respiratory events.

**Main Points**

• Evaluate and contrast the use of dexmetatomidine versus propofol-fentanyl for painless sedation in patients undergoing colonoscopy.

• To check the effectiveness and safety of both sedation modalities.

## Introduction

Sedation is used during colonoscopy procedures for patients who experience difficulty remaining calm due to issues such as anxiety, pain, or physical discomfort.^1^ Various anaesthetic techniques have been employed for colonoscopy. These techniques encompass both awake and asleep periods, with or without mechanical ventilation, as well as the management of patients who remain fully conscious throughout the procedure.^2^ The level of sedation required can vary from patient to patient, but the patient must remain adequately sedated and pain-free during the process.^2^ Anaesthesiologists have employed various intravenous sedative drugs to induce conscious sedation or provide monitored anaesthesia care during colonoscopies, with many using a combination of short-acting opioids such as remifentanil and propofol.^3^

Dexmetatomidine is a highly selective and potent α2-adrenoceptor agonist that has sedative, pain killer, anxiolytic, opioid-sparing, and sympatholytic effects.^4^ In contrast to alternative sedatives, α2 adrenergic receptor agonists do not induce respiratory depression.^4^ The expected pharmacokinetics and brief half-life within 5-6 min following loading dose injection enable the implementation of titration to achieve a favorable effect.^4^ The sedative characteristics of dexmetatomidine are expressed through the hyperpolarization of noradrenergic neurons in the locus coeruleus.^5^ Dexmetatomidine exerts its sedative effects by inducing hyperpolarization of noradrenergic neurons in the locus coeruleus.^5^ This particular form of sedation, known as “combining sedation”, may be beneficial for colonoscopy procedures requiring a deep level of sedation, ensuring adequate patient cooperation.^5^

This research aims to evaluate and compare the use of dexmetatomidine versus propofol-fentanyl for painless sedation in patients undergoing colonoscopy for various indications. We hypothesized that dexmedetomidine can be used as a solo agent for conscious sedation in colonoscopy procedures without or with minimal affection of hemodynamics or the need for airway intervention to replace the routine practice with propofol and fentanyl.

## Methods

The current study participants were recruited from different hospitals of Al-Azhar University from December 2021 to May 2023. A total of 60 eligible patients of both sexes, aged between 21 and 60 years, with an American Society of Anesthesiologists (ASA) I and II, were randomized into two groups.

This study was approved by the Ethical Committee of the Department of Anaesthesiology, Intensive Care and Pain Management at Al-Azhar University (Registration number: 00385/2023). The study was registered in ClinicalTrials.gov under the number NCT06148103.

All patients provided informed written consent. The research was conducted under the revised Helsinki Declaration of the World Medical Association and the International Council for Harmonisation of Technical Requirements for Pharmaceuticals for Human Use Good Clinical Practice guidelines for rigorous clinical study practice.

Group D received dexmedetomidine, whereas Group PF received propofol-fentanyl infusions in equal ratios. The initial dose of dexmedetomidine was 1 µg kg^-1^ over 10 min, and then a maintenance infusion was titrated in a range from 0.2-1 µg kg^-1^ h^-1^). The administration of propofol was maintained at a rate of 25 to 150 mg h, whereas fentanyl infusion was maintained at 0.01 to 0.05 µg kg^-1^ min^-1^. The doses of all drugs were modified to achieve a target level of sedation ranging between 2 and 4 points on the modified Observer’s Assessment of Alertness/Sedation (OAA/S) scale.^6^

**Type of study:** A prospective, randomized, double-blind trial.

### Primary Outcome

The primary outcome of this study was to assess the efficacy of painless sedation during colonoscopy. The patient’s ability to collaborate and perform the procedure was evaluated using a 10-point numerical rating scale (NRS).

### Secondary Outcomes

⦁  High occurrence of adverse effects such as airway obstruction, respiratory depression, and hemodynamic insecurity.

⦁  Failure to provide adequate sedation.

### Randomization

Participants were allocated to either the D or PF groups using a simple randomization procedure. The allocation sequence was generated by a single researcher who ensured blinding of the allotment process by using sequentially sealed, opaque, and numbered envelopes. Another researcher implemented the randomization process and recruited patients.

A researcher who was blinded to the study procedures was responsible for collecting all intra- and postoperative data. We blinded the patients to their group allotment. Two infusion syringe pumps were employed for each patient, and measures were taken to conceal the infusion syringes and extension lines to prevent identification.

### Anaesthetic Procedure

The patients were placed in the supine position, ensuring their comfort. Vital parameters were measured using standard monitors, including electrocardiography, non-invasive blood pressure monitoring, and pulse oximetry (SpO_2_). All patients exhibited spontaneous respiration and were administered oxygen at a flow rate of 4 l min via simple nasal prongs. The end-tidal carbon dioxide monitor was connected to the oxygen nasal prongs to monitor the patient’s respiratory rate (RR).

Following the insertion of a venous peripheral line, the research infusions were initiated following both drug sedation protocols. In case of anxiety, pain, or restlessness during the procedure, the rate of infusion of dexmetatomidine (Group D) or propofol-fentanyl (Group PF) was markedly increased. Inadequate sedation in either group was addressed by increasing the infusion rates initially, with the backup of a propofol stat dose of 20 to 30 mg intravenously given if first-plane treatment was unsuccessful. 10 min before the procedure, propofol infusion was stopped, and both dexmetatomidine and fentanyl infusions were downgraded. Minimum infusion rates of both fentanyl (0.01-0.05 µg kg^-1^ min^-1^) in the PF group and dexmetatomidine (0.1-0.4 µg kg^-1^ h^-1^) in the D group were extended during colonoscopy, which lasted approximately 45 min.

We observed patients in the post-anaesthesia care unit (PACU) after completion of the procedure for 2 h before being released into the ward. All basic monitoring procedures were performed during their time in the PACU. In cases where nausea and vomiting occurred during the postoperative period, patients were administered ondansetron 4 mg and/or metoclopramide 20 mg intravenously as necessary. Once discharged from the PACU, the gastroenterology team decided on pain management and hospital discharge.

### Measurements

⦁  The patient’s ability to collaborate and perform the procedure was evaluated using a 10-point NRS, with a score of 8 or higher indicating a successful colonoscopy.

⦁  During colonoscopy, we assessed the level of sedation using the modified OAA/S scale. In addition, we requested patients to evaluate their anxiety levels at 12 specific intervals using visual analog scales (VAS).

⦁  We asked candidates to grade degrees of pain: 0 (no); 1 to 3 (mild); 4 to 6 (moderate); 7 to 10 (severe pain) and anxiety: 0 to 1 (no or mild); 2 to 3 (moderate); 4 to 5 (severe anxiety).

⦁  This grading was repeated at 12 subsequent times during colonoscopy: T0 (baseline), T1 (starting of the procedure), T2 (5 min after T1), and then 5-min intervals from T3 to T9. T10 (finishing off the procedure); T11 (patient admittance to PACU); and T12 (after T11 by 120 min.).

⦁  After 24 h of colonoscopy, patients were interviewed individually to inquire about any potential adverse events they might have encountered, such as nausea, vomiting, or pain.

⦁  Patients were asked if they would be willing to undergo the same anaesthetic technique again if necessary. Telephone conversations were organized in cases where patients had been cleared from the hospital on the day of the procedure.

### Sample Size Justification

A discrepancy of 25% in the ability to perform a successful colonoscopy was deemed clinically significant. To determine a significant difference of 2.5 grading on the NRS scale for colonoscopy between the D and PF categories, it was deemed appropriate to have a sample size of 30 participants per group, resulting in 60 participants. This decision was made on the basis of a dual-sided analysis with a significance level (α) of 0.05, a statistical power of 90%, a standard deviation of 1, and accounting for a 10% potential dropout rate.

### Statistical Analysis

Statistical analysis was performed using the SAS analytical program, variant 9.3 (SAS establishment, Cary, NC, USA]. All investigations were performed on an altered motive-to-treat set, which included all participants with a basic parameter throughout the clinical evaluation. We used Wilcoxon rank-sum analysis to establish a correlation between steady variables and univariate differences in the D and PF groups. In addition, the χ^2^ test was used for categorical variables. Consecutive data were expressed as median (25-75% interquartile range) or mean [standard deviation (SD)], and group variables were expressed as count (%).

The levels of pain, anxiety, and sedation were compared between the two groups using one-way analysis of variance (ANOVA). A revised analysis of variance (ANOVA) was performed to evaluate the fluctuations of HR, mean arterial pressure (MAP), SpO_2_, and RR during the procedure. The least-squares mean discrepancies between both groups were correlated, and the corresponding 95% confidence intervals (CI) and *P* values were provided. A significance level of *P*<0.05 was examined.

## Results

### Participant Characteristics

**Figure 1:**
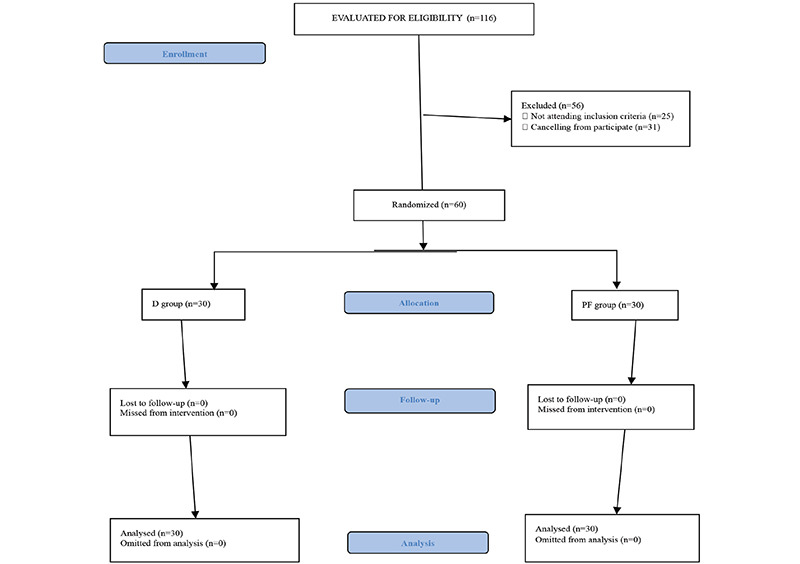
Consort flow graph.

From October 2021 to May 2023, 116 patients underwent screening to determine their eligibility for the study. Before randomization, 56 patients were excluded, resulting in 60 patients who were equally divided into two groups, as shown in the consort flow chart (Figure 1): the D group (n=30) and the PF group (n=30). Significantly, there were no participants who were lost during the study.

Table 1 presents baseline patient characteristics and clinical features. There were no significant differences between the D and PF groups regarding patient demographic data, ASA physical status, co-morbidities, and anaesthesia time.

### Outcome Variables

**Figure 2:**
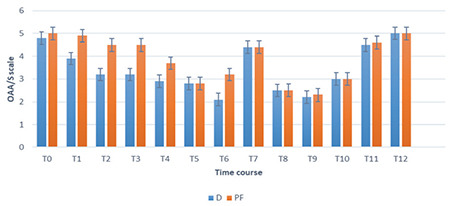
Modified Observer’s Assessment of Alertness/Sedation rate.

The colonoscopy procedure, performed with effective sedation, yielded positive results in all participants, as indicated by an overall mean NRS score (SD) of 8.95 (0.51) and a range of 7-10. The study found that the ability to perform colonoscopy under sedation was comparable between the D and PF groups. The mean NRS scores (94% CI) were 9.8 (9.6-10.0) for the D group and 9.3 (9.1-10.0) for the PF group. The *P* value was 0.15. The mean OAA/S scores (94% CI) were 3.8 (3.4-5.1) and 4.8 (3.3-5.5) for the D and PF groups, respectively, with a *P* value of 0.49 (Figure 2).

**Figure 3:**
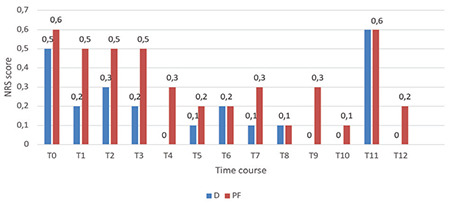
Numerical rating scale for anxiety. NRS anxiety was checked at successive times (T0 to T12). Infusions of the drug were initiated at T0 and ended at T10. Outcomes are expressed as means (SD). D category is dexmedetomidine; PF category is propofol-fentanyl; T0, baseline before procedure start; T1, starting colonoscopy; T2, 5 min gap after T1; T3-T9; 5 min gaps; T10, colonoscopy finish; T11, shift to PACU; T12, after shifting to PACU by 120 min. *P < 0.05.

**Figure 4:**
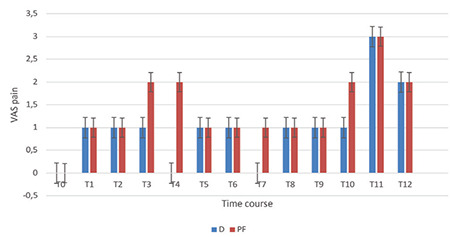
Visual analog scale for pain. VAS pain was checked at successive times (T0-T12). Infusions of the drug were initiated at T0 and ended at T10. Outcomes are expressed as means (SD). D category is dexmedetomidine; PF category is propofol-fentanyl; T0, baseline before procedure start; T1, starting colonoscopy; T2, 5 min gap after T1; T3-T9; 5 min gaps; T10, colonoscopy finish; T11, shift to PACU; T12, after shifting to PACU by 120 min. *P < 0.05.

The OAA/S scores during colonoscopy were significantly lower in the D group at time points T1-T12 (*P*=0.043) (Figure 2). The duration of awakening after the infusions ended was similar in both groups, lasting approximately 4-9 min. No notable disparity was observed in the NRS scores for anxiety between the groups throughout the procedure, as depicted in Figure 3. Group D demonstrated significantly reduced pain VAS scores during the T4 (*P*=0.030) and T7 (*P*=0.029) intraoperative time points, as shown in Figure 4.

**Figure 5:**
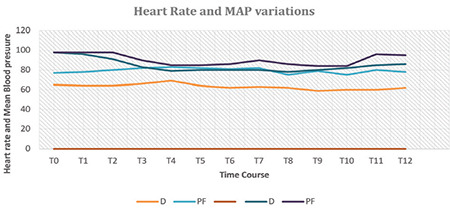
Heart rate and mean arterial blood pressure varieties during colonoscopy. The spectrum of documented readings for mean arterial pressure in the D group was 60-105 mmHg; the spectrum for mean arterial pressure in the PF group was 55-100 mmHg. Infusions of the drug were initiated at T0 and ended at T10. Replicated readings ANOVA revealed that heart rate was significantly low [mean difference (94% CI): -14.3 (-18.4, -9.1) beats min^-1^, P < 0.001] in the dexmedetomidine group. The dimension of documented readings for HR in the dexmedetomidine group was 38-112 beats/min; ranges for HR in the PF group were 44-148 beats min^-1^. Infusions of the drug were initiated at T0 and ended at T10. Outcomes are expressed as means (SD). D category is dexmedetomidine; PF category is propofol-fentanyl; T0, baseline before procedure start; T1, starting colonoscopy; T2, 5 min gap after T1; T3-T9; 5 min gaps; T10, colonoscopy finish; T11, shift to PACU; T12, after shifting to PACU by 120 min. *P < 0.05.

**Figure 6:**
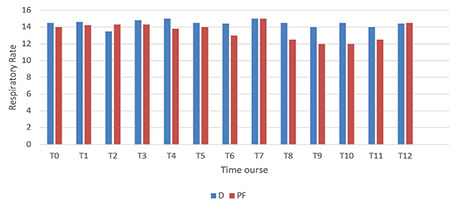
Respiratory rate alterations during colonoscopy. The dimensions of documented readings for RR in the dexmedetomidine group were 6-26 cycles/m; ranges for RR in the PF group were 7-27 cycles/m. Infusions of the drug were initiated at T0 and ended at T10. Outcomes are expressed as means (SD). D category is dexmedetomidine; PF category is propofol-fentanyl; T0, baseline before procedure start; T1, starting colonoscopy; T2, 5 min gap after T1; T3-T9; 5 min gaps; T10, colonoscopy finish; T11, shift to PACU; T12, after shifting to PACU by 120 min. *P < 0.05.

**Figure 7:**
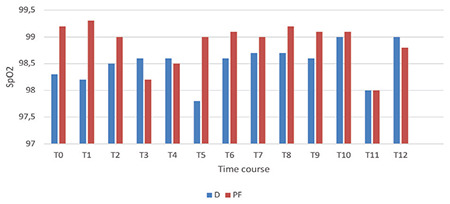
SpO_2_ alterations during colonoscopy. The dimension of documented readings for oxygen saturation (SpO_2_) in the dexmedetomidine group was 85-99%; the dimension of documented readings for oxygen saturation (SpO_2_) in the PF group was 89-100%. Infusions of the drug initiated at T0 and ended at T10. Outcomes are expressed as means (SD). D category is dexmedetomidine; PF category is propofol-fentanyl; T0, baseline before procedure start; T1, starting colonoscopy; T2, 5 min gap after T1; T3-T9; 5 min gaps; T10, colonoscopy finish; T11, shift to PACU; T12, after shifting to PACU by 120 min. *P < 0.05.

Figures 5, 6, 7 display the measurements of hemodynamic parameters. Group D exhibited a noticeable decrease in MAP during the intraoperative period at times T6 to T8, with corresponding *P* values of 0.024, 0.006, and 0.024, respectively. The values at T11 and T12 were substantially low. It was also significantly low at T11 and T12, admittance to PACU with *P* < 0.001, and 30 min following admission to PACU (*P*=0.004). A significant interaction between time and both groups were observed for mean arterial pressure (MAP), with a *P* value of 0.044 (Figure 5). Repeated measures ANOVA analyses demonstrated a statistically significant decrease in heart rate during the procedure in Group D [mean difference (96% CI): -13.7 (-19.2, -8.6) beats min^-1^, *P* < 0.002] over the procedure time in Group D, as demonstrated in Figure 5. In the PF group, RRs were significantly low at T8 with a *P* value of 0.030 and T10 with a* P* value of 0.002, as depicted in Figure 6. There was a significant difference in SpO_2_ between both groups throughout the procedure (Figure 7).

Table 2 presents the prevalence of adverse events during the intraoperative period. The prevalence of pulmonary adverse effects requiring interference was low in group D compared with the PF group (0% vs. 23% respectively) (*P*=0.023).

Cardiovascular complications included transient hypotension managed with low-dose ephedrine (n=3) or phenylephrine (n=2), transient hypertension managed with labetalol (n=2) and hydralazine (n=1), a brief episode of bradycardia managed with atropine, and some agitation or emotional upset managed with a low dose of midazolam.

**Figure 8:**
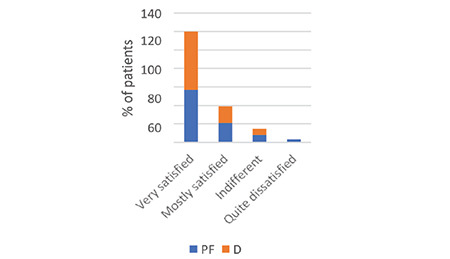
Patient satisfaction

**Figure 9:**
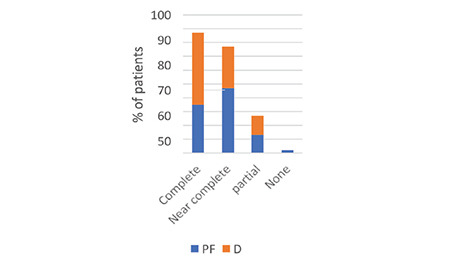
Experience recall

**Figure 10:**
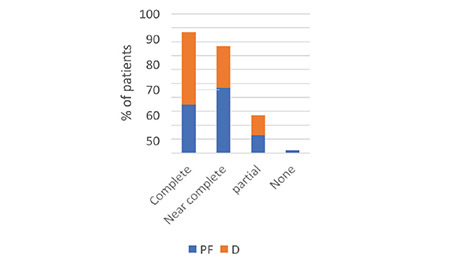
Anxiety recall

The assessment of patient satisfaction and memory of the colonoscopy procedure was conducted 24 h after the procedure using the Likert scale. During a structured meeting, we requested participants to assess their experience with the procedure by completing the following questionnaire: (1) overall satisfaction with comfort level, (2) intraprocedural recall, and (3) recall of the degree of intraoperative discomfort and anxiety, as indicated in Figures 8, 9, 10. All patients’ inputs were significantly biased toward the D group.

## Discussion

In this comparative, randomized, prospective, and double-blind study, the use of either dexmedetomidine- or propofol-fentanyl-based sedation during colonoscopy yielded a similar intraprocedural efficacy. There was no difference in the occurrence of cardiovascular or other negative effects between the two groups. Furthermore, the prevalence of respiratory adverse effects was significantly higher in the propofol-fentanyl group. The levels of perioperative anxiety, pain, patient satisfaction, and recall were similar in both groups.

Compared with propofol-fentanyl, the administration of dexmetatomidine resulted in a decline in heart rate during the procedure time and a decline in mean arterial pressure throughout the minimal provocative times. However, the decline in heart rate did not exceed 20% from baseline.

Utilizing propofol for sedation, typically in conjunction with short-acting opioids, is a highly effective method for sedating patients during colonoscopy,^7^ leading to a high level of patient satisfaction.^8^ Nevertheless, irrespective of the selected anaesthetic method, colonoscopy remains a challenging procedure for patients who suffer from anxiety.^9^ An optimal sedative medication should have extensive therapeutic evidence and anticipated pharmacodynamics to ensure adequate sedation.^10^ Dexmedetomidine exhibits a synchronized sedation pattern, enabling patients to transition quickly from a state of sleepiness to wakefulness, follow instructions while conscious, and return to sleep when not stimulated.^11^ The use of propofol for colonoscopy procedures was more satisfactory than dexmedetomidine infusions to patients in the Kavousi et al.^2^ study, but on the other hand, there were recordings of respiratory depression in many patients who needed respiratory and airway support. This negative point will be reflected in the sedation doses needed to keep the patient well sedated, which might be increased. Our protocol is different from the Kavousi et al.^2^ 2021 protocol in that we started dexmetatomidine infusion throughout the procedure, which helped to keep the patient well sedated during the procedure, but they started dexmetatomidine as a stat dose only. Consecutive research analyzing the effect of dexmetatomidine on the capability to perform intraoperative neurologic evaluation has yielded conflicting results.^12^

Recently, a comparison was performed between dexmedetomidine with midazolam and midazolam alone for procedural sedation during awake fiberoptic tracheal intubation.^13^ Dexmetatomidine was determined to be as efficient when given with midazolam as midazolam alone.^13^ In our study, we continued dexmetatomidine alone for the dexmetatomidine group throughout the procedure, which helped for a steady state level of sedation and a shorter awakening time after the procedure. The shorter awakening times observed in recent studies can be attributed to the comparatively lower levels of sedation administered before colonoscopy and the relatively brief duration of the procedure.

A single anaesthetic agent may not be effective for all phases of colonoscopy. The preparatory aspect of the colonoscopy procedure can be highly irritating. During this component, the patient may need further analgesia and sedation. Ensuring that patients remain free from anxiety during this procedural period is of utmost importance.^13^

In our study, we assessed the level of sedation using the OAA/S scale. Although this scoring method is subjective and relies on analytic information, the OAA/S scale is a dependable and valid tool with minimal variability between different raters. Previous research has also demonstrated a strong correlation between bispectral index during dexmetatomidine and propofol sedation and the OAA/S scale.^14^

For our dosing of both dexmedetomidine and fentanyl-propofol, we did not find any significant variations in heart rate or mean blood pressure, which may be related to our use of glycopyrrolate prophylactically to prevent bradycardia and extrasystoles in some patients. Our findings coincide with the results of Karanth et al.^10^. They evaluated an initial dose of propofol 2-3 mg kg^-1^ over 10 min followed by a continuous infusion of 25 µg kg^-1^ min^-1^ in one group and compared it to another group where they started a bolus dose of dexmetatomidine 1 µg kg^-1^ intravenously over 10 min followed by a maintenance infusion of 0.2 µg kg^-1^ h^-1^ until the end of the procedure. They did not find any significant difference between the two groups regarding the vital parameters. However, we used fentanyl in addition to propofol to add an analgesic benefit.

## Conclusion

The administration of dexmetatomidine and propofol-fentanyl during colonoscopy was found to be safe. Nevertheless, dexmetatomidine may present notable benefits in this context because of its lower occurrence of respiratory adverse events. Achieving an ideal dosage regimen of sedatives, along with maintaining a vigilant approach, are crucial factors for ensuring successful conscious sedation during colonoscopy.

## Figures and Tables

**Table 1:**
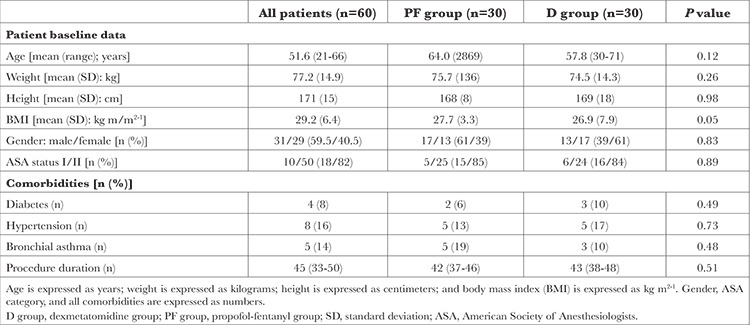
Baseline Data and Duration of Colonoscopy in All Patients

**Table 2:**
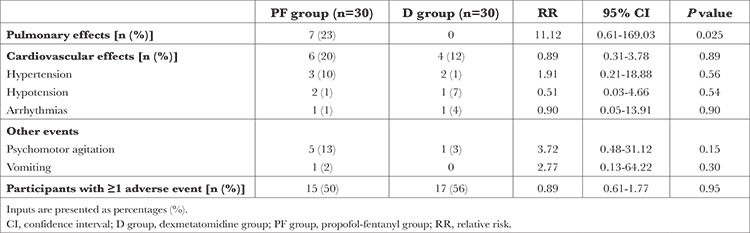
Prevalence of Intraprocedural Adverse Events
